# Inherited Developmental and Epileptic Encephalopathies

**DOI:** 10.3390/neurolint13040055

**Published:** 2021-11-03

**Authors:** Emanuele Bartolini

**Affiliations:** USL Centro Toscana, Neurology Unit, Nuovo Ospedale Santo Stefano, 59100 Prato, Italy; emanuele.bartolini@uslcentro.toscana.it

**Keywords:** epilepsy, genetics, intellectual disability

## Abstract

Epileptic encephalopathies often have a genetic etiology. The epileptic activity itself exerts a direct detrimental effect on neurodevelopment, which may add to the cognitive impairment induced by the underlying mutation (“developmental and epileptic encephalopathy”). The focus of this review is on inherited syndromes. The phenotypes of genetic disorders affecting ion channels, metabolic signalling, membrane trafficking and exocytosis, cell adhesion, cell growth and proliferation are discussed. Red flags suggesting family of genes or even specific genes are highlighted. The knowledge of the phenotypical spectrum can indeed prompt the clinician to suspect specific etiologies, expediting the diagnosis.

## 1. Introduction

An epileptic encephalopathy (EE) is a severe type of epilepsy in which the epileptic activity itself promotes cognitive and behavioural impairments above and beyond what might be expected from the underlying pathology alone [1].

Cognitive functions may be slowed, plateauing or regress, and patients often develop psychiatric and behavioural disturbances. A key component of the EE concept is that amelioration of the epileptiform activity may have the potential to improve the developmental consequences of the disorder [2]. We usually consider EE to exclusively occur in the paediatric population. Nevertheless, cognitive impairment may occur at any age and the concept of EE may be also applicable to syndromes arising in adulthood.

A genetic aetiology is often to be sought. A polygenic or complex genetic background can be involved, and modifier genes and epigenetics factors can also partially influence the phenotype [3].

However, in recent years, techniques of next-generation sequencing (NGS) applied to severe epilepsies have allowed to identify a growing number of single gene mutations that can promote seizures and affect neurodevelopment. Strikingly, some genetic syndromes may be characterized by early waning of epilepsy but persisting profound developmental consequences at long-term. Often it may not be possible to disentangle whether the epileptic or developmental component plays the major role.

To navigate through the nosological boundaries of these syndromes, different descriptors can be applied: “developmental encephalopathy” (DE), where there is just developmental impairment without frequent epileptic activity; “epileptic encephalopathy” (EE), where there is no pre-existing developmental delay, and the genetic mutation is not thought to cause slowing per se; and “developmental and epileptic encephalopathy” (DEE), where both factors play a role. To disclose a specific genetic aetiology in these syndromes is very important. Families are often relieved to find a cause after a distressing diagnostic conundrum; unnecessary testing can be avoided, and appropriate familial counselling can be established.

Most severe epilepsies result from de novo mutations, with a low recurrence risk due to germline mosaicism when parents are unaffected (estimated to be about 1%). The possibility of parental somatic mosaicism in apparent de novo variants have also to be considered, with reported figures up to 10% of families. The resulting recurrence risk cannot be calculated, but can approach 50% as observed in autosomal dominant disorders [4,5]. Familial clustering can also result from parental transmission with classical mendelian inheritance, especially for genes with variable penetrance and expressivity [6].

The spotlight of the current review will be on severe epilepsies in which the epileptic activity itself affects neurodevelopment (EE/DEE), especially focusing on inherited syndromes. For this purpose, the phenotype of inherited mutations affecting ion channels, metabolic signalling, membrane trafficking and exocytosis, cell adhesion, and cell growth and proliferation will be discussed.

## 2. Methods

The literature search was performed on the Medline and Embase databases. Articles published in English up to August 2021 were sought using Boolean logic with the following search terms: ‘inherited AND epil *’, ‘inherited AND encephalopathy’.

**Figure 1:**
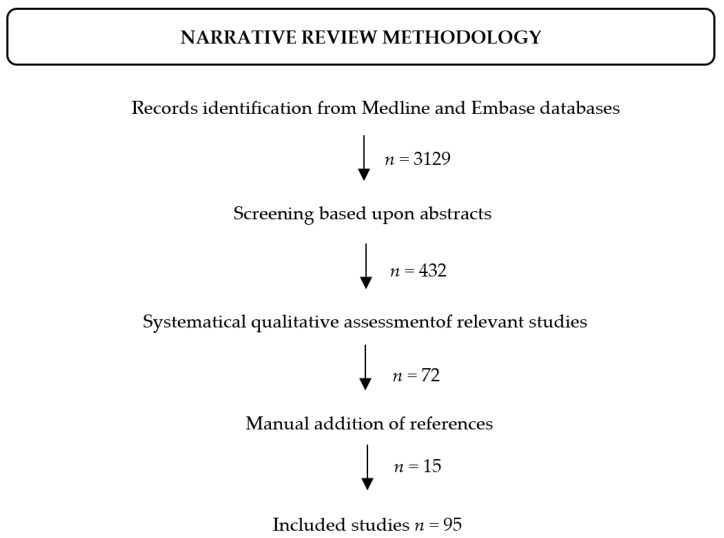
Flow-chart depicting the literature review process.

The retrieved articles have first been screened based upon the abstracts. The full text of those deemed to be relevant have then been systematically assessed. Additional references have been added after manual search on PubMed, Embase, the Human Gene Mutation Database (HGMD, http://www.hgmd.cf.ac.uk/ac/index.php), Google (https://www.google.com) and Google Scholar (https://scholar.google.com) search engines (accessed on 30 October 2021) (Figure 1).

## 3. Channelopathies

Ion channels are proteins located across cell membranes to form a pore through which ions move according to their electrochemical gradient.

Each channel exhibits a selective permeability to specific ions and is usually composed by a pore-forming subunit and accessory regulatory subunits (heteromeric structure). Channel gating can be regulated mostly by transmembrane electric potential (*voltage-gated channels*) or specific extracellular ligand molecules (*ligand-gated channels*).

Genetic channelopathies can promote systemic disorders, consistent with the ubiquitous expression of ion channels. The central nervous system is especially vulnerable, considering ion channels affect the generation, suppression, and spread of the action potential [7]. Most phenotypical traits are characterized by paroxysmal symptoms, especially seizures. Epilepsy may arise at any age [8] according to the different expression of ion channels during development [9].

Classical epilepsy syndromes (Table 1) and specific syndromes associated with single ion channel genes are well described.

The vast majority of genetic channelopathies have initially been reported in families with benign epilepsies. However, the large-scale application of NGS techniques has revealed the genotype–phenotype correlation may be extremely challenging. Ion channel genes initially associated only with benign phenotypes (e.g., *KCNQ2*, *SCN2A*) have also been demonstrated to be implicated in severe EE/DEE.

As a rule of thumb, inherited variants mostly provoke familial benign syndrome, and de novo mutations promote EE/DEE. There are remarkable exceptions: cases of EE/DEE can cluster within a family or co-occur with milder epilepsies as expression of variable expressivity.

### 3.1. Voltage-Gated Channels

Inherited EE/DEE have been reported to result from disruption of several voltage-gated channels.

Voltage-gated sodium channels (‘VGSCs’ or ‘Nav channels’) play a major role. A superfamily of genes clustering on chromosome 2 encodes for different isoforms of the pore-forming alpha subunit of this channel (Nav1.1–Nav1.9), whose opening results in a sodium inflow [10].

Mutations affecting three alpha subunit genes (*SCN1A*, *SCN2A*, and *SCN8A*) have been shown to cause epilepsies of variable severity [11].

*SCN1A* encodes for the alpha 1 subunit (Nav1.1), whose expression steadily augments from 4–5 months of age on [12]. The phenotypical spectrum of *SCN1A* mutations spans from mild to severe epilepsies, all characterized by fever-sensitive seizures. Dravet syndrome is the classical encephalopathic phenotype and mostly result from de novo heterozygous mutations (truncating, splice-site, frameshift intragenic deletions, gene duplications leading to haploinsufficiency, missense mutations affecting the pore-forming region of the protein) [13,14].

Inherited autosomal dominant mutations occur in 5–10% of cases and are usually missense. The syndrome can also be inherited for somatic or germline mosaic mutations in unaffected parents, with familial clustering in the offspring [13,15].

Dravet syndrome typically arises suddenly with unilateral and very prolonged febrile seizures at 5–8 months of age in previously healthy children [16]. This time window coincides with the period of mandatory vaccination in children; these patients were formerly considered to suffer an alleged ‘vaccine encephalopathy’ before the underlying *SCN1A* neurobiology was discovered [17,18]. During the first year of life, both febrile and afebrile seizures occur. From the second year of age, neurodevelopment plateaus and seizures flare as generalized tonic-clonic, alternating unilateral clonic, focal with impaired awareness, brief myoclonic, tonic (rare) or myoclonic non-convulsive status epilepticus described as ‘obtundation status’ [19,20,21]. Sodium-channel-blocker antiseizure medications (ASMs) (e.g., carbamazepine, phenytoin, lamotrigine) may precipitate seizures. The EEG background activity worsens over time, with generalized spike-wave and polyspike-wave activity, multifocal discharges, and sometimes photosensitivity [19,22]. Autistic traits and language impairment may establish themselves, and a wide-based ataxic gait sets in. After five years of age, epilepsy wanes with less frequent seizures [19,21,23]. In adulthood, myoclonic and absence seizures disappear; tonic/convulsive seizures occur mainly in sleep, and ataxia, dysarthria, intention tremor, and intellectual disability prevail [24,25]. Levodopa-responsive extrapyramidal signs and axonal motor neuropathy may also ensue [26].

The *SCN2A* gene encodes for the Nav1.2 channel, which is highly expressed from the birth [27]. Mutations of *SCN2A* have been increasingly recognized as a major cause of EE/DEE [16,28]. De novo missense and truncating variants are those usually associated with severe phenotypes. Inherited variants are mostly associated with milder epilepsies (e.g., benign familial neonatal-infantile seizure, autosomal dominant, missense mutations). Intrafamilial phenotypical heterogeneity is possible but rare. Cases of EE/DEE and mild epilepsy in the same family or febrile seizures are described suggesting variable expressivity [16]. In patients with *SCN2A*-related encephalopathy, seizures arise either from the first week of life or during childhood, accompanied by cognitive regression and autistic features [16,29]. The onset is abrupt, with focal seizures and spasms in clusters, possibly leading to specific syndromes such as Epilepsy of Infancy with Migrating Focal Seizures (EIMFS) and Ohtahara syndrome [16]. Recent findings suggest neonatal-onset seizures are provoked by gain-of-function missense mutations and respond to sodium-channel-blocker ASMs, whilst later onset epilepsies are associated to loss-of-function missense or truncating variants and do not respond these type of medications [29].

Heterozygous missense mutations of *SCN8A* have recently been associated with a wide spectrum of epilepsies. Inherited mutations almost exclusively yield benign infantile epilepsies; however SCN8A-related EE/DEE due to parental germline mosaicism has been described [30,31,32]. The *SCN8A* gene product (sodium channel subunit Nav1.6) is increasingly expressed from the neonatal period, progressively replacing the Nav1.2 channel in initial axonal segments. Accordingly, most patients with *SCN8A* mutations develop epilepsy in infancy (median age = 4 months) [32]. Patients with *SCN8A*-related encephalopathy suffer global developmental delay, sleep disorders, frequent startles and multiple intractable seizure types (focal, tonic with autonomic signs, clonic, myoclonic, absence, epileptic spasms) [32,33,34]. Dystonic/dyskinetic attacks may also occur [30,32].

Potassium channelopathies can also be responsible of EE/DEE, especially those affecting the voltage-gated ion channels (Kv) regulating the frequency and duration of action potential [35], such as those encoded by *KCNQ2* and *KCNQ3*.

Mutations of these genes were initially identified in patients with autosomal dominant benign familial neonatal seizures [36,37]. Like many other channelopathies, the phenotypic spectrum now also includes severe epilepsies, including families with drug-resistant seizures and intellectual disability harboring inherited mutations of *KCNQ2* [38] and *KCNQ3* [39].

*KCNQ2*-related encephalopathy is characterized by drug-resistant tonic asymmetric, focal and clonic seizures in the first week of life, progressively waning in early infancy and remitting by the age of 1–3 years. The corresponding electroencephalographic patterns are neonatal burst-suppression evolving to multifocal epileptiform activity during follow-up. Sodium channel blockers ASMs can be effective. Early seizure freedom can ameliorate the neurodevelopmental outcome [40]. A dominant negative effect of *KCNQ2* mutations has been demonstrated. The vast majority of mutations are missense and appear to cluster in four putative functional hotspots, affecting the conformation of the tetrameric subunits of the channel [41].

KCNQ3-related encephalopathy is similarly characterized by early onset drug-resistant seizures, centrotemporal EEG abnormalities, and intellectual disability at long-term [39].

Mutations affecting inwardly rectifying potassium (Kir) channels can provoke EE/DEE, especially missense mutations of *KCNB1* disrupting the voltage sensor and the pore domain. The corresponding phenotype is characterized by predominant language difficulties, behavioral impairment, and seizures in 85% of cases. A single case with an inherited truncating variant has been identified in this syndrome (p.Arg583 *) [42].

Specific severe epileptic phenotypes can also result from the *KCNT1* gene. Inherited missense mutations were initially reported in an unusual form of autosomal dominant sleep-related hypermotor epilepsy (SHE) complicated by intellectual disability and behavioural disturbances. Sporadic mutations mainly yield EIMFS: nearly continuous multifocal and migrating seizures in the first 6 months of life followed by persistent drug-resistant epilepsy with acquired microcephaly, intellectual disability, axial hypotonia, pyramidal, and extrapyramidal signs. However, both cases of SHE and EIMFS can co-occur within the same family. Irrespectively of the underlying syndrome, up to 30% of patients benefit from quinidine, an anti-arrhythmogenic potassium-channel blocker, for seizure control [43,44].

Eventually, calcium channelopathies may also be responsible of EE/DEE. These channels regulate the inflow of calcium ions according to their electrochemical gradient modulating gene transcription, neurotransmitter release, neurite outgrowth, and enzyme activity [45]. Heterozygous mutations affecting *CACNA1A* are those most often encountered in neurological phenotypes. This gene is involved in hemiplegic migraine, alternating hemiplegia, and episodic ataxia, both sporadic and familial. Different phenotypical traits may overlap in a *CACNA1A* family: each family member can exhibit seizures, episodes of hemiplegia, migraine, or ataxic disorders with variable penetrance and expressivity. Some degree of intellectual disability can also occur [7,46,47,48,49]. With regard to epilepsy, two main alternative phenotypes have been identified in these patients (i) early and recurrent status epilepticus followed by drug-resistant focal-onset epilepsy, early progressive cerebellar signs, hemiplegic accesses with consciousness impairment, severe intellectual disability (ii) generalized epilepsy with early-onset refractory absence seizures, stable cerebellar dysfunction, and milder intellectual disability [7].

Biallelic *CACNA1A* mutations inherited from unaffected parents have been disclosed in probands with early onset seizures, intellectual disability, autistic features, and progressive cerebral and optic nerve atrophy [50].

### 3.2. Ligand Gated Channels

Genetic dysfunction of GABA receptors can promote EE/DEE. *GABRA1A* is indeed a major causative gene for various epileptic encephalopathies, mostly sporadic but occasionally inherited through autosomal dominant mutations. Patients may develop Ohtahara syndrome, infantile spasms, Dravet syndrome-like phenotypes and myoclonic astatic epilepsy [51]. Red flags for suspecting *GABRA1A* mutations are infantile-onset epilepsy (from 1 day to 15 months of age), prominent tonic-clonic and myoclonic seizures, generalized spike-and-wave activity and photoparoxysmal response. No clear genotype-phenotype correlation has emerged, although the clinical presentation appears to be quite homogeneous within single families [51].

The ligand-gated NMDA receptor is a glutamate-dependent ion channel. Mutations of *GRIN2A* and *GRIN2B*, encoding for two subunits of the receptor, can yield several neurodevelopmental disorders and be inherited in an autosomal dominant manner. The phenotypical spectrum of *GRIN2A* mutations include isolated intellectual disability, idiopathic focal epilepsy, and epileptic encephalopathies. Language disorders are common and range from mild speech impairment with no seizures to the epilepsy aphasia spectrum (atypical rolandic epilepsy, continuous spikes and waves during slow-wave sleep, and Landau–Kleffner-Syndrome) [52]. Gain-of-function *GRIN2B* mutations cause West syndrome, as well as childhood onset focal epilepsy in association with intellectual disability. Cognitive skills depend upon the degree of channel function impairment [53].

## 4. Metabolic Disorders

Seizures and intellectual disability can also result from inborn errors of metabolism (IEM), often following an autosomal recessive pattern. Some of these disorders can be resolutely addressed with specific treatments (treatable IEM). A timely diagnosis may indeed limit or even prevent the devolvement of an overt encephalopathy as well as suggest investigating extra-cerebral comorbidities. Although the phenotype may be extremely complex, the clinician should be prompted to suspect metabolic disorders when encountering red flags such as dysmorphic features, organomegaly, positive family history and/or consanguinity, ophthalmological disorders, and metabolic acidosis with high anion gap. From the neurological standpoint, cognitive regression with worsening myoclonic seizures should ring a bell. Seizures may be triggered by external factors such as periods of stress, intercurrent illnesses, or metabolic complications (e.g., hypoglycemia) [54,55,56].

Seizures may also arise abruptly with unspecific characteristics, especially in the newborn. In this age range, we should especially investigate Pyridoxine dependency epilepsy and Pyridox(am)ine 5′phosphate oxidase (PNPO) deficiency.

Pyridoxine-dependent epilepsy begins with multifocal drug-resistant seizures since the first hours/days of life, namely, myoclonic jerks, tonic seizures, and focal motor seizures. It is provoked by autosomal recessive *ALDH7A1* mutations. The biochemical defect consists in deficiency of α–aminoadipic semialdehyde dehydrogenase (antiquitin), which is involved in cerebral lysine metabolism. Patients should be promptly treated by pyridoxine to interrupt seizures and hamper cognitive deterioration (vitamin B6:100 mg intravenously, followed by oral supplementation 30 mg/kg/day in two divided doses for 3–7 days). Indeed, a trial of pyridoxine is recommended in any newborn with stormy onset seizures of unknown etiology given the dramatic change of prognosis it may bring. Diagnostic confirmation is done through demonstration of elevated levels of intermediary substrates in urine and/or plasma and cerebrospinal fluid [56,57].

Deficit of PNPO may give a similar phenotype. This enzyme is essential for the synthesis of pyridoxal phosphate (PLP), which is the active form of pyridoxin. These patients and those newborns not responding to pyridoxine should be expeditely treated by PLP supplementation. The definitive diagnosis can only be established by molecular genetic testing (autosomal recessive inheritance) [58].

In older children, a progressive EE/DEE can result from defects of cerebral glucose transporter (GLUT1). These are caused by mutations of *SLC2A1* gene, mostly inherited as autosomal dominant or due to sporadic haploinsufficiency. Cases of autosomal recessive inheritance have been described.

The phenotype ranges from severe early onset drug-resistant seizures, developmental delay, and acquired microcephaly (De Vivo syndrome) to early onset absence epilepsy or Epilepsy with Myoclonic-Atonic Seizures [59]. Clinical clues to GLUT1 deficiency include an increase in seizures during fasting, progressive cognitive impairment, and paroxysmal exercise-induced dyskinesia. The diagnosis can result either from the finding of low CSF glucose levels or from molecular genetic testing. When suspecting GLUT1 deficiency, ketogenic diet should be introduced as this non-pharmacological treatment limits both seizures and neurological deterioration by providing alternative sources of energy to the brain (ketone bodies) [59].

Many other IEM can provoke seizures and intellectual disability at different ages with variable expressivity. For instance, neuronal ceroid-lipofuscinoses may affect the central nervous system of newborns, toddlers, children, or even adults, inducing progressive intellectual deterioration, vision impairment without organomegaly, and epilepsy that may follow a progressive myoclonic course [60,61]. The inheritance pattern can be autosomal dominant or recessive. A comprehension of the underlying molecular biology can have a striking impact on patient management. In 2019, a patient-customized rescue therapy was rapidly developed for a 6-year-old child with neuronal ceroid lipofuscinosis 7, after disclosing a pathogenic variant in the gene *MFSD8* (inherited from the father) and a cryptic second hit affecting splice-site in intron 6 of the same gene (inherited from the mother). Researchers developed an antisense oligonucleotide (milasen) to block the splice-site mutation and obtained an objective improvement in seizure control and cognitive functioning [62].

Mitochondrial disorders similarly can arise at different ages and promote seizures with intellectual disability. The proteins of mitochondria are m encoded by nuclear DNA (nDNA) and in a small percentage by the mitochondrial DNA (mtDNA). Both mutations of nDNA (autosomal dominant or recessive) and mtDNA (maternally inherited) can affect the respiratory chain and the oxidative phosphorylation. Mitochondrial disorders tend to involve multiple organs with high energy demand (e.g., nervous system, skeletal and cardiac muscles, liver, kidneys, and endocrine system).

For instance, POLG-related disorders (nDNA) are characterized by progressive neurodegeneration, refractory epilepsy, movement disorder, neuropathy, and hepatic failure; seizures can be myoclonic, prolonged tonic-clonic, or focal motor (epilepsia partialis continua). Valproate is absolutely contraindicated [63,64].

Pyruvate dehydrogenase complex deficiency can result from mutations of different nDNA genes (*PDHA*, *PDHB*, *LIAS*, *LIPT1*, *DLD*, and *PDH*). In infancy, infantile spasms, clonic seizures, or refractory focal epilepsy can be observed, as well as developmental delay, ataxia, hypotonia, hypertonia, abnormal eye movements, dystonia, and axonal neuropathy; ketogenic diet may partially be effective in seizure control [65,66].

Leigh syndrome has a wide genotypical heterogeneity (>90 nDNA or mtDNA genes) and is typically characterized by focal drug-resistant seizures, developmental regression/delay, and basal ganglia/brainstem abnormalities on neuroimaging [67].

Myoclonic epilepsy with ragged red fibres (MERFF) (*MTTL1* (80%): m.8344A > G; *MTTK* (10%): m.8356T > C, m.8363G > A, m.8361G > A) provoke an adult-onset progressive myoclonic epilepsy. Seizures can also often be generalized tonic, clonic or atonic. Cerebellar ataxia, cardiac arrhythmias, myopathy, diabetes, hearing loss, and dementia ensue [66,68].

Mitochondrial encephalopathy, lactic acidosis, and stroke-like episodes (MELAS) (*MTTL1* gen: m.3243A >G m.3271T > C; *MTND5* gen: m.13513G > A) are mostly characterized by seizures during a stroke-like episode, often with focal status epilepticus and secondary encephalopathy [66,69].

To summarize, identifying a specific metabolic disorder in subjects with such complex phenotypes can be challenging. A tiered approach to the diagnosis of IEM can be usefully applied. A preliminary screening by history and physical exam and wide spectrum blood and urine biochemistry should be performed, after which targeted biochemical and molecular investigations can be undertaken [56].

## 5. Membrane Trafficking and Exocytosis

Genes involved in transmembrane trafficking may directly and indirectly affect neuronal excitability. Like channelopathies, the corresponding phenotypes span from benign to very severe epilepsies.

Mutations of *STX1B* are a relevant example. The *STX1B* gene encodes for syntaxin-1B, which composes the SNARE complex together with SNAP25 and synaptobrevin. This complex mediates the process of calcium-dependent synaptic vesicle release to enable neurotransmitter exocytosis and also interacts with the syntaxin binding protein 1 protein (encoded by *STXBP1*) [70].

Variants of *STX1B* provoke fever-associated epilepsies of variable severity including Epilepsy with Myoclonic-Atonic Seizures [71,72]. Intrafamilial clustering due to autosomal dominant mutations with variable expressivity also including EE/DEE has recently been reported [73].

Another gene involved in membrane trafficking is *SYNGAP1*, which regulates the postsynaptic density at excitatory glutamatergic neurons through the cytosolic protein SYNGAP1 (SYNaptic GTPase Activating Protein). Loss-of-function mutations causing haploinsufficiency lead to intellectual disability and severe epilepsy. This syndrome has indeed only been reported as sporadic, but a single inherited case has been due to parental mosaicism [74].

## 6. Cell Adhesion

The disruption of intercellular adhesion is especially detrimental for brain function.

Protocadherin 19 is a crucial molecule in this regard. It is a calcium dependent cell-cell adhesion molecule encoded by the *PCDH19* gene, highly expressed during brain development, and involved in neuronal migration and in the establishment of synaptic connections. The gene is located on chromosome Xq22.3. Heterozygous mutations of *PCDH19* typically cause a particular EE/DEE characterized by a singular mode of inheritance. Only heterozygous females are affected (X-linked dominant pattern). Hemizygous males are asymptomatic but can transmit the pathogenic variant to daughters. Mosaic males can exhibit complete or incomplete phenotype.

The condition results from loss-of-function variants causing cellular interference: in affected females and mosaic males, the presence of both wild-type and mutant cells interfere with one another due to the production of different surface proteins, whilst non-mosaic hemizygous males produce a homogenous population of cells [75,76,77]. Cases resulting from de novo mutations are also reported.

Typical features of PCDH19-related encephalopathy include generalized or focal seizures highly sensitive to fever, intellectual disability, autistic traits. Epilepsy may explode with brief recurrent seizure clusters (generally lasting 1–5 min and repeating multiple times a day) in late infancy lasting through childhood possibly leading to intensive care unit. The phenotype can resemble Dravet syndrome and according to some studies, *PCDH19* variants are found in 25% of *SCN1A*-negative females exhibiting features of Dravet syndrome [75,78].

## 7. Cell Growth and Proliferation

The vast majority of the aforementioned EE/DEE exhibit a normal brain MRI or unspecific findings. Conversely, mutations affecting genes involved in cell growth and proliferation can disrupt brain morphology.

Mutations in the Aristaless-related homeobox gene (*ARX*) may promote an encephalopathic phenotype with either normal imaging (X-linked West syndrome, X-linked myoclonic epilepsy with spasticity and intellectual disability, Partington syndrome with mental retardation, ataxia and dystonia, and nonsyndromic forms of mental retardation) or associated to brain malformations (X-linked lissencephaly with abnormal genitalia). ARX-related conditions are inherited in an X-linked recessive manner or can be sporadic. Female carriers are generally asymptomatic or have mild phenotypes. Hence, it is always appropriate to test a seemingly unaffected mother of an affected proband, and skewed X-inactivation can occur [79,80].

The ARX protein helps to regulate the process by which cells differentiate and migrate. ARX-knockout mice are defective in neuroblast proliferation and in GABAergic interneuron migration [79,81].

Similarly, mutations of other genes involved in neurodevelopment can provoke malformative EE/DEE. Lissencephaly is the most common radiological pattern, but there are also peculiar MRI abnormalities that can suggest the involvement of specific genes (Table 2). Most mutations are de novo, yet familial cases are described as autosomal dominant (*LIS1*, *DYNC1H1*, tubulin-encoding *TUBA1A*, *TUBB2A*, *TUBB2B*, *TUBB3*, *TUBB [TUBB5]*, and *TUBG1* genes), X-linked dominant (*DCX*), or autosomal recessive (tubulin-encoding *TUBA8*).

Tubulinopathies may also have coloboma, heart defects, ichthyosiform dermatosis, mental retardation, and ear anomalies with hearing loss.

In most cases, global developmental delay is associated to epileptic spasms in the infancy, often followed by drug-resistant epilepsy in the long-term [82,83,84].

The gene *CDKL5* is another important target of mutations provoking EE/DEE and exerts its functions within the AKT/GSK-3β signaling pathway, which is fundamental in processes of brain development, namely, neuronal precursor proliferation, survival, and maturation [87]. Mutations in CDKL5 are associated with a severe X-linked EE/DEE characterized by severe intellectual disability, generalized developmental delay, early-onset intractable seizures, infantile spasms, and Rett’s syndrome (RTT)-like features. The phenotype shares striking similarities with RTT: hyperventilation, hand stereotypies, and hypotonia affecting young females [88,89]. The majority of RTT cases are heterozygous for missense or nonsense mutations in the gene encoding for methyl-CpG-binding protein 2 (MeCP2) [90]. Several CDKL5 gene mutations, resulting in missense, nonsense, splice, and frameshift mutations or genomic deletions have been described in girls (heterozygous mutations) and a few boys [88]. In females, the phenotypic spectrum of the disease ranges from milder forms, which include the possibility of autonomous walking and drug-responsive seizures, to severe EE/DEE. In males, the phenotype is always severe [91].

Abnormalities in cell growth and proliferation can also lead to multisystem disorders. An outstanding example is Tuberous Sclerosis Complex (TSC), a neurocutaneous syndrome characterized by early onset EE/DEE and extracerebral involvement. Most cases are sporadic, about one third instead result from inherited autosomal dominant mutations. The causative genes are *TSC1/TSC2*, respectively encoding tuberin and hamartin, both of which physiologically inhibit the oncogenic mTOR pathway. Pathogenic mutations of these genes result in over-activation of the mTOR within all TSC-associated lesions yielding hamartomatous lesions and an increased risk of tumors.

Most children develop West syndrome. A specific ASM (vigabatrin) has been shown to bring a specific efficacy, but drug-resistance often accrues. The cognitive outcome is unfavorable, due to the presence of multiple malformations of cortical development (cortical tubers, subependymal nodules) and tumors (subependymal astrocytomas) that can render TSC patients surgical candidates. Extracerebral typical features are cutaneous (hypomelanotic macules, shagreen patches, angiofibromas), renal (renal cysts, hamartomas, angiomyolipomas), cardiac (rhabodmyoma), and pulmonary (lymphangioleiomyomatosis).

The mTOR inhibitor everolimus have been shown to reduce the size of renal and brain lesions and improve pulmonary function in TSC, as well as effectively decrease seizure frequency [92,93].

## 8. Conclusions

The clinical spectrum of the inherited EE/DEE range is very wide. Different mutations can apparently provoke common severe epilepsy syndromes. Next-generation sequencing techniques have revolutionized the landscape of molecular biology allowing a wide-spectrum screening of patients with EE/DEE. Despite this, the clinician’s assessment remains of paramount importance as suspecting a specific diagnosis can prompt appropriate testing and accelerate treatment. A punctual knowledge of the phenotypical spectrum can indeed constitute solid support to ameliorate the prognosis of patients and to accurately counsel family members.

## Figures and Tables

**Table 1:** Classical epilepsy syndromes corresponding to EE/DEE (modified from https://www.epilepsydiagnosis.org/) (accessed on 30 October 2021).

	Clinical Overview	Typical Age at Onset
Early Myoclonic Encephalopathy	Myoclonic seizures are frequent, distinguishing this syndrome from Ohtahara syndrome	1–3 months
Ohtahara syndrome	Tonic seizures predominate, myoclonic seizures are uncommon	1–3 months
West syndrome	Epileptic spasms, hypsarrhythmia and global developmental delay	3–12 months
Dravet syndrome	Normal child with prolonged, febrile and afebrile, focal and tonic-clonic seizures at onset	Around 6 months of age
Epilepsy Of Infancy With Migrating Focal Seizures	Focal seizures arise independently in both hemispheres and can migrate from one cortical region to another randomly but consecutively in the same seizure.	First 6 months
Epilepsy With Myoclonic-Atonic Seizures	Myoclonic-atonic seizures in an otherwise normal child who may have a history of febrile and/or afebrile seizures.	6 months–6 years
Epileptic Encephalopathy With Continuous Spike-And-Wave During Sleep	Focal seizures, atypical absences, negative myoclonus/atonic seizures and neurocognitive regression	2–12 years
Landau-Kleffner syndrome	Subacute onset of acquired aphasia. Seizures may not occur in all cases and when present are infrequent and self-limiting	2–8 years

**Table 2:** Genes commonly associated to malformation of cortical development observed in families [85,86].

Affected Gene	Main Imaging Phenotypes
*ARX*	X-linked lissencephaly with abnormal genitalia
*DCX*	Anteriorly predominant lissencephaly (males) Subcortical band heterotopia (females)
*LIS1*	Posteriorly predominant lissencephaly
*TUBA1A*	Posteriorly predominant lissencephaly ± absent corpus callous, and cerebellar hypoplasia; polymicrogyria like pattern
*TUBB2B*	Posteriorly predominant lissencephaly ± cerebellar hypoplasia; polymicrogyria like
*TUBB3*	Absent corpus callosum, polymicrogyria like pattern
*TUBB [TUBB5]*	Absent corpus callosum, polymicrogyria like pattern
*TUBG1*	Posteriorly predominant lissencephaly
*DYNC1H1*	Posteriorly predominant lissencephaly

## Data Availability

Not applicable.
